# A novel FcγRIIa-TRIM54-STAT2 axis negatively regulates type I interferon signaling and promotes viral susceptibility in human monocytes

**DOI:** 10.1128/jvi.02106-25

**Published:** 2026-05-29

**Authors:** Xiao-Qiu Dai, Shenghao Hua, Lian Xue, Zheng Gong, Ya-Ying Pan, Zhenjun Li, Chaojie Han, Xiao-Ming Gao, Fang-Yuan Gong

**Affiliations:** 1 School of Basic Medical Sciences, Soochow University 12582, Suzhou, China; 2 Department of Clinical Laboratory, Children’s Hospital of Soochow University 12582, Suzhou, China; 3 Department of Rheumatology, Kowloon Hospital 545455, Suzhou, China; University of Virginia, Charlottesville, Virginia, USA

**Keywords:** IFN-I, TRIM54, STAT2, monocyte, virus infection

## Abstract

While adaptive humoral immunity and type I interferon (IFN-I) responses are known to cross-regulate each other during viral infection, the mechanisms by which antibodies (Abs) or immune complexes (ICs) modulate IFN-I signaling in immune cells remain unclear. Here, using plate-coated human IgG (cIgG) as an antigen-independent IC mimic, we show that FcγRIIa cross-linking in human blood monocytes induces the expression of TRIM54, an E3 ubiquitin ligase previously thought to be constitutively expressed only in muscle tissue. Mechanistically, TRIM54 binds to and promotes the ubiquitination of the transcriptional activator STAT2, thereby dampening the expression of many IFN-stimulated genes. This inhibitory role is functionally corroborated by a positive correlation between elevated TRIM54 levels and enhanced cellular permissiveness to infection by vesicular stomatitis virus, Dengue virus, and/or Sendai virus, in cIgG-primed human monocytes, and monocytic cell lines with genetically modulated TRIM54 expression. Clinically, elevated TRIM54 expression is detected in circulating monocytes from pediatric patients with Epstein-Barr virus-induced infectious mononucleosis. Together, our findings define a previously unrecognized FcγRIIa–TRIM54–STAT2 signaling axis through which virus-specific Abs—particularly at sub-neutralizing concentrations—can attenuate the IFN-I response in innate immune cells. This axis may also provide a mechanistic explanation for Ab-dependent enhancement of viral infection.

**IMPORTANCE:**

The long-standing question of how antigen-antibody immunocomplexes regulate type I interferon (IFN) signaling in immune cells remains unanswered. Our study uncovers a novel pathway for negative regulation of IFN-I signaling via the FcγRIIa-TRIM54-STAT2 axis in human monocytes. The novel concept will provide new insights into cross-regulation between humoral immunity and IFN response, and may provide important clues for the molecular mechanisms behind antibody-dependent enhancement of virus infection.

## INTRODUCTION

The type I interferon (IFN-I) family represents an important first line of defense against virus infections in humans. They are secreted by host cells upon detection of viral components via cellular pattern-recognition receptors (PRRs) such as Toll-like receptors (TLRs), RIG-I-like receptors (RLRs), and cytosolic DNA sensors (CDSs), and are able to function in both autocrine and paracrine fashions. IFN-Is comprise multiple IFN subtypes, of which IFN-α (13 homologous subtypes) and IFN-β are the best defined and most broadly expressed (1). They can induce an antiviral state in virus-infected as well as uninfected bystander cells by activating the IFN-stimulated genes (ISGs) through the IFN-α receptor 2 (IFNRA2), Janus kinase (JAK), and signal transducer and activator of transcription (STAT) signaling cascade (1–3). Regulation mechanisms for IFN-I production and function are of great interest to the fields of medical immunology and IFN biology (2–4).

Following IFN-dominated immediate action against viral invasion, the immune system launches adaptive humoral (antibody, Ab) and cellular responses, mediated by B and T lymphocytes, respectively, for more precise and durable protection. It is generally believed that there is continued cross-regulation between the adaptive humoral immunity and IFN-I response during viral infections. For example, IFN-Is suppress the production of neutralizing Abs in mouse models of chronic lymphocytic choriomeningitis virus (LCMV) infection through IFN-I-mediated killing of neutralizing Ab-producing B cells (5–7). On the other hand, adaptive humoral responses coincide with a strong and rapid decline of IFN-Is initially in blood and subsequently in local tissues following viral infection *in vivo*. Remarkably, however, the questions of whether and how Abs, or Ab-virus immunocomplexes (ICs), regulate IFN-I production and/or signaling in immune cells remain so far elusive.

In the present study, we demonstrate that human FcγRIIa crosslinking by plate-coated human IgG (cIgG, a mimic IC without antigen restriction) strongly blocks IFN-I signaling and consequently enhances virus permissiveness in human monocytes via induction of tripartite motif protein 54 (TRIM54), an E3 ubiquitin protein ligase constitutively expressed in muscles (8–11). Our results uncover a novel FcγRIIa-TRIM54-STAT2 pathway for IC-mediated regulation on IFN-I signaling in human innate immune cells, and may provide useful clues for the molecular mechanisms behind Ab-dependent enhancement (ADE) of virus infection, which occurs when ICs of sub-neutralizing Abs and virus particles interact with FcγRs on monocytes, dendritic cells (DCs), and macrophages (12, 13).

## RESULTS

### Solid-phase IgG downregulates IFN-I signaling for ISG transcription in human primary blood monocytes

**Fig 1:**
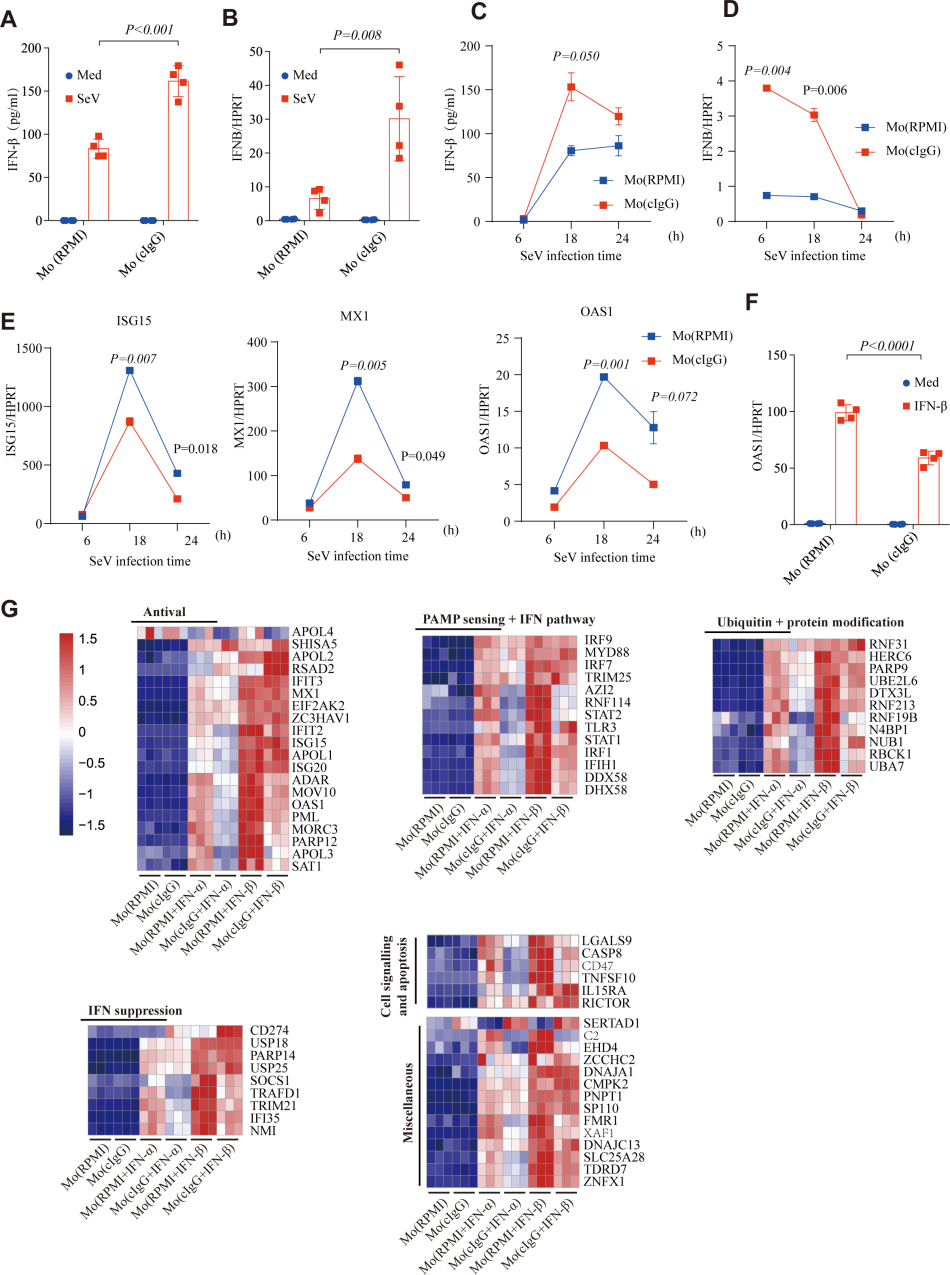
cIgG promotes IFN-I production but suppresses ISG activation in human monocytes. (**A, B**) Freshly fractionated human CD14^+^ blood monocytes from heathy volunteers were precultured in the presence [Mo(cIgG)s] or absence [Mo(RPMI)s] of cIgG for 24 h, and then stimulated with or without (Med) SeV for 18 h, followed by ELISA quantitation of IFN-β in culture supernatant and Q-PCR detection of *IFNB* transcription. (**C, D**) Time courses of the IFN-β production and *IFNB* transcription of Mo(cIgG) and Mo(RPMI) cells responding to SeV stimulation. (**E**) Mo(cIgG) and Mo(RPMI) cells were stimulated with SeV for 6, 18, and 24 h, followed by Q-PCR detection of *ISG15, MAX1*, and *OAS1* transcription. (**F**) Mo(cIgG) and Mo(RPMI) cells were stimulated with or without IFN-β in triplicate wells for 18 h, followed by Q-PCR detection of *OAS1* transcription. PCRs were performed using *HPRT* as internal control. The pretreated cells were stimulated with SeV or IFN-b in triplicate wells, and the data are shown as mean ± SD. These are representatives of three independent experiments using blood samples from different healthy volunteers. (**G**) Samples of Mo(RPMI)s and Mo(cIgG)s from three unrelated healthy individuals were stimulated for 12 h with or without IFN-α or IFN-β and then subjected to high throughput RNA sequencing analysis. Heatmaps show relative transcription of the 62-vertebrate core ISGs in the six groups of monocytes.

In a previous work we reported that FcγR crosslinking by cIgG primes human monocytes toward a hyperresponsive state, enhancing TNF-α response to bacterial lipopolysaccharide (LPS) (14). Here, we ask whether cIgG-trained human monocytes also exhibit heightened IFN-I responsiveness upon viral stimulation. Freshly isolated CD14^+^ human monocytes were pretreated with cIgG and subsequently stimulated with Sendai virus (SeV). Compared with control monocytes cultured in medium alone [Mo(RPMI)], cIgG-primed human monocytes [Mo(cIgG)] secreted significantly more IFN-β and showed elevated *IFNB* transcription in response to SeV (Fig. 1A through D), confirming that cIgG-licenses IFN-β hyper-responsiveness. Given that IFN-Is function in both paracrine and autocrine fashions, we expected that virus-stimulated Mo(cIgG) cells would display enhanced ISG activation. Surprisingly, however, SeV-induced transcription of ISGs, such as *OAS1, MX1*, and *ISG15* in Mo(cIgG) cells, was significantly lower than in Mo(RPMI) cells (Fig. 1E), which is further corroborated by reduced *OAS1* transcription in Mo(cIgG) cells responding to IFN-β (Fig. 1F). These data suggest that cIgG priming renders monocyte resistance to IFN-I-driven ISG activation. RNA sequencing analysis results of the 62 vertebrate core ISGs (15) further confirmed widespread ISG suppression in IFN-α or IFN-β-stimulated Mo(cIgG) cells (Fig. 1G). Of note, while *MX1* and *ISG15* suppression was clearly evident in Mo(cIgG) cells treated with IFN-α, it was less pronounced with IFN-β stimulation (Fig. 1G, antiviral panel), which may reflect the particularly strong potency of the IFN-β used in these assays. *RSAD2, APOL2,* and *SHISHA5* were the only unsuppressed “antiviral” ISGs in Mo(cIgG) cells stimulated with either IFN-α or IFN-β. We conclude that cIgG triggers two temporally overlapping but functionally distinct signaling outcomes in human monocytes: it enhances IFN-I production while simultaneously suppressing the downstream IFN-I signaling required for activation of most ISGs.

### Induction of TRIM54 expression in human monocytes by IgG-IC through FcγRIIa signaling

**Fig 2:**
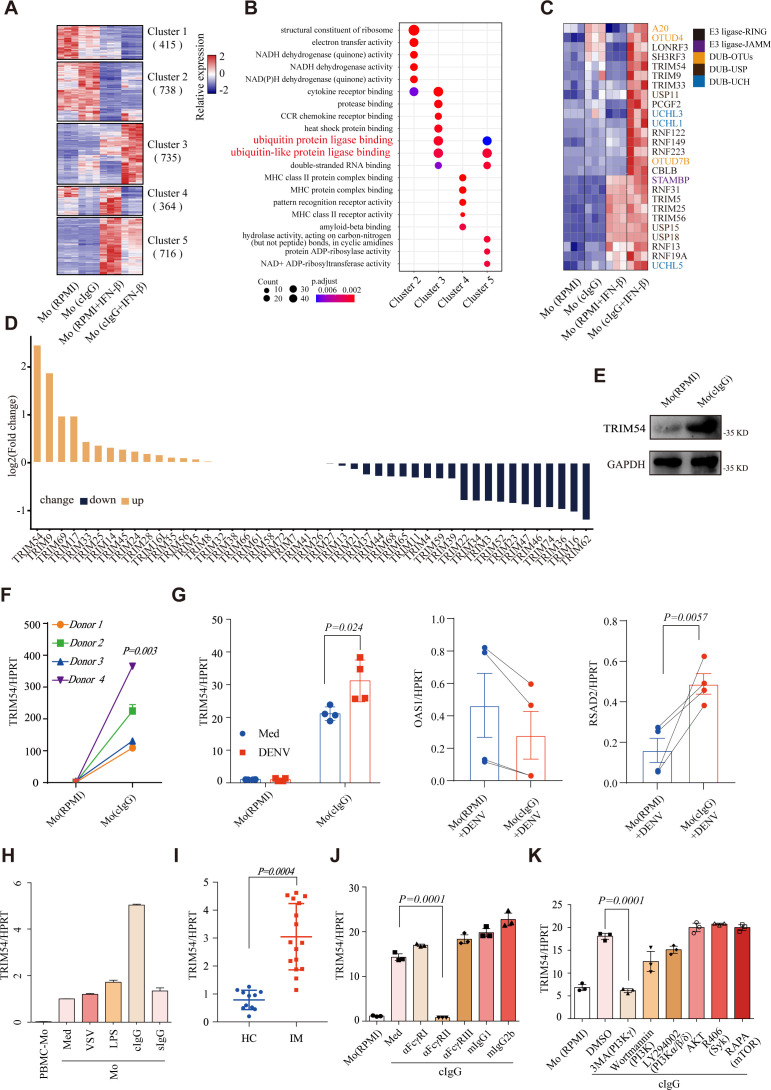
Induced TRIM54 expression in human monocytes by FcγRIIa crosslinking. (**A**) K-means (*K*  =  5) clustering of 2,968 differentially expressed genes in any pairwise comparison among four conditions. Clusters are indicated on the left. (**B**) Dot-plot showing the adjust *P*-value of GO term enrichment for genes in Clusters 2–5. (**C**) Heatmap showing the relative expression of differential E3 ligase and deubiquitinating enzymes in response to IFN-β stimulation. (**D**) Differential expression of TRIM genes in Mo(cIgG)s vs Mo(RPMI)s as revealed by transcriptomic analysis. The results are expressed as *fold change* of Mo(cIgG)s compared with Mo(RPMI)s. (**Ε**) Lysates of Mo(cIgG)s and Mo(RPMI)s were subjected to WB using HRP-labeled Abs against human TRIM54 (upper blot) or GAPDH (lower blot) as probes. (**F**) Fractionated blood monocytes from four healthy donors (Donors 1–4) were cultured in the presence [Mo(cIgG)] or absence [Mo(RPMI)] of cIgG for 24 h. (**G**) Human monocytes that had been pretreated with [Mo(cIgG)] or without [Mo(RPMI)] cIgG for 24 h were further cultured in the presence or absence (Med) of DENV (MOI: 1) for 12 h. The prepared cells were subjected to Q-PCR detection of TRIM54, OAS1, and RSAD2 transcription, using HPRT as internal control. (**H**) PBMCs from healthy subjects were fractionated into monocytes (Mo) and monocyte-depleted (PBMC-Mo) populations. Monocytes were cultured for 24 h in the presence or absence (Med) of soluble IgG (sIgG), cIgG, LPS, or VSV, while PBMC-Mo cells were unstimulated. (**I**) Circulating monocytes were freshly fractionated from peripheral blood samples of EBV-infected pediatric patients (blood cell EBV load > 10^4^/mL) with infectious mononucleosis (IM, *n* = 14) and age- and gender-matched uninfected control subjects (HC, *n* = 20). (**J**) Human blood monocytes were stimulated with cIgG for 24 h in the presence or absence (Med) of blocking Abs against human FcγRI (FcγRI), FcγRII (FcγRII), or FcγRIII (FcγRIII). Irrelevant mouse IgG1 (mIgG1) and IgG2b (mIgG2b) were included as isotype controls. Untreated cells maintained in R10 medium alone [Mo(RPMI)] were included as negative control. (**K**) Human monocytes were stimulated with cIgG for 24 h in the presence of chemical inhibitors RAPA (100 nM), R406 (100 nM), 3MA (1 mM), AKT (10 nM), LY294002 (100 nM), or Wortmannin (100 nM). DMSO was included as solvent control. Untreated cells maintained in R10 medium alone [Mo(RPMI)] were included as negative control. (**H–K**) The prepared cells were subjected to Q-PCR detection of TRIM54 transcription using HPRT as internal control, and the results are expressed as mean TRIM54/HPRT ± SD. The experiments were repeated at least three times using primary monocytes freshly isolated from PBMCs of unrelated healthy donors with consistent results. The data shown are from one representative experiment.

To elucidate the molecular mechanisms underlying ISG suppression in cIgG-preconditioned human monocytes, we analyzed differentially expressed genes (DEGs) between Mo(cIgG) and Mo(RPMI) cells following IFN-β stimulation. Of the 2968 DEGs, only one cluster (Cluster 3) comprising 735 genes was upregulated by IFN-β in Mo(cIgG) cells (Fig. 2A). Gene ontology (GO) analysis of the Cluster 3 genes highlighted two dominant functional categories: regulation of monocyte/macrophage chemokine/cytokine secretion and ubiquitination-related posttranslational modification (Fig. 2B and C). The latter group includes several genes encoding E3 ubiquitin ligases—such as A20, TRIM54, TRIM9, and TRIM33—which are known to regulate antiviral immunity and IFN responses (4, 16–20). This prompted us to analyze the TRIM family expression profile in cIgG-treated myocytes using our previously deposited transcriptomic data (14). As shown in Fig. 2D, TRIM54 and TRIM9 were the only two TRIM genes showing >1-fold increased transcription in blood monocytes after cIgG stimulation, TRIM33 also ranked among the top five upregulated TRIM genes. Although TRIM9 was originally regarded as brain-specific, recent studies indicate its expression in macrophages and its role in regulating innate immune signaling via NF-κB and IRF3 (19, 20). TRIM54, previously characterized as a constitutively expressed E3 ligase in muscle tissues (8–11), has also been reported in human liver cancer cells and rat tendon-derived stem cells (21–23); however, its expression and function in immune cells remained unknown.

In this study, we obtained multiple lines of evidence demonstrating IC-inducible TRIM54 expression in human monocytes: (i) strong TRIM54 protein signal by Western blot in Mo(cIgG) but not in Mo(RPMI) cells (Fig. 2E); (ii) detection of TRIM54 mRNA by Q-PCR in cIgG-stimulated—but not unstimulated—peripheral blood monocytes from independent healthy donors (Fig. 2F); (iii) virus (DENV) stimulation significantly further enhanced TRIM54 expression in cIgG-preconditioned, but not unstimulated, human monocytes (Fig. 2G); and (iv) unlike cIgG, neither soluble IgG (sIgG) nor potent PAMP activators, such as LPS and virus particles (VSV, SEV) alone, could induce TRIM54 expression in human monocytes *in vitro* (Fig. 2H). Beyond *in vitro* evidence, TRIM54 expression was also observed *in vivo*. Q-PCR analysis of circulating monocytes from pediatric patients with Epstein-Barr virus (EBV)-associated infectious mononucleosis (EBV load >10⁴/mL) revealed TRIM54 transcripts in most patient samples (*n* = 14), whereas none were detected in age- and gender-matched uninfected controls (*n* = 20) (Fig. 2I).

Human monocytes express three different types of activating FcγRs: FcγRIa, FcγRIIa, and FcγRIIIa (12, 13). Our earlier works identified FcγRIIa as the principal receptor mediating cIgG-induced cytokine hyperresponsiveness or osteoclast differentiation of human monocytes (14, 24). Consistent with this, FcγR-blocking experiments confirmed that FcγRIIa is required for cIgG-induced TRIM54 expression (Fig. 2J). Using kinase inhibitors targeting PI3Kγ (3MA), PI3Kα/β/δ (LY294002), pan-PI3K (Wortermanin), mTOR (RAPA), AKTi (MK-2206 2HCl), or Syk (R406), we found that PI3Kγ-specific inhibitor 3MA most potently suppressed TRIM54 induction, with Wortmannin showing moderate inhibition at 100 nM (Fig. 2K). The weak effect of Syk inhibitor R406 was notable, as Syk and PI3K are known to act in parallel in FcγRIIa signaling in human monocytes and neutrophils (25). Perhaps PI3Kγ activation downstream of FcγRIIa crosslinking is sufficient to initiate TRIM54 expression. Taken together, these data demonstrate that cIgG triggers FcγRIIa-dependent TRIM54 induction in human monocytes via PI3Kγ, and that TRIM54 expression can be further amplified by viral stimulation.

### TRIM54-mediated regulation of IFN-I signaling in monocytic cells

**Fig 3:**
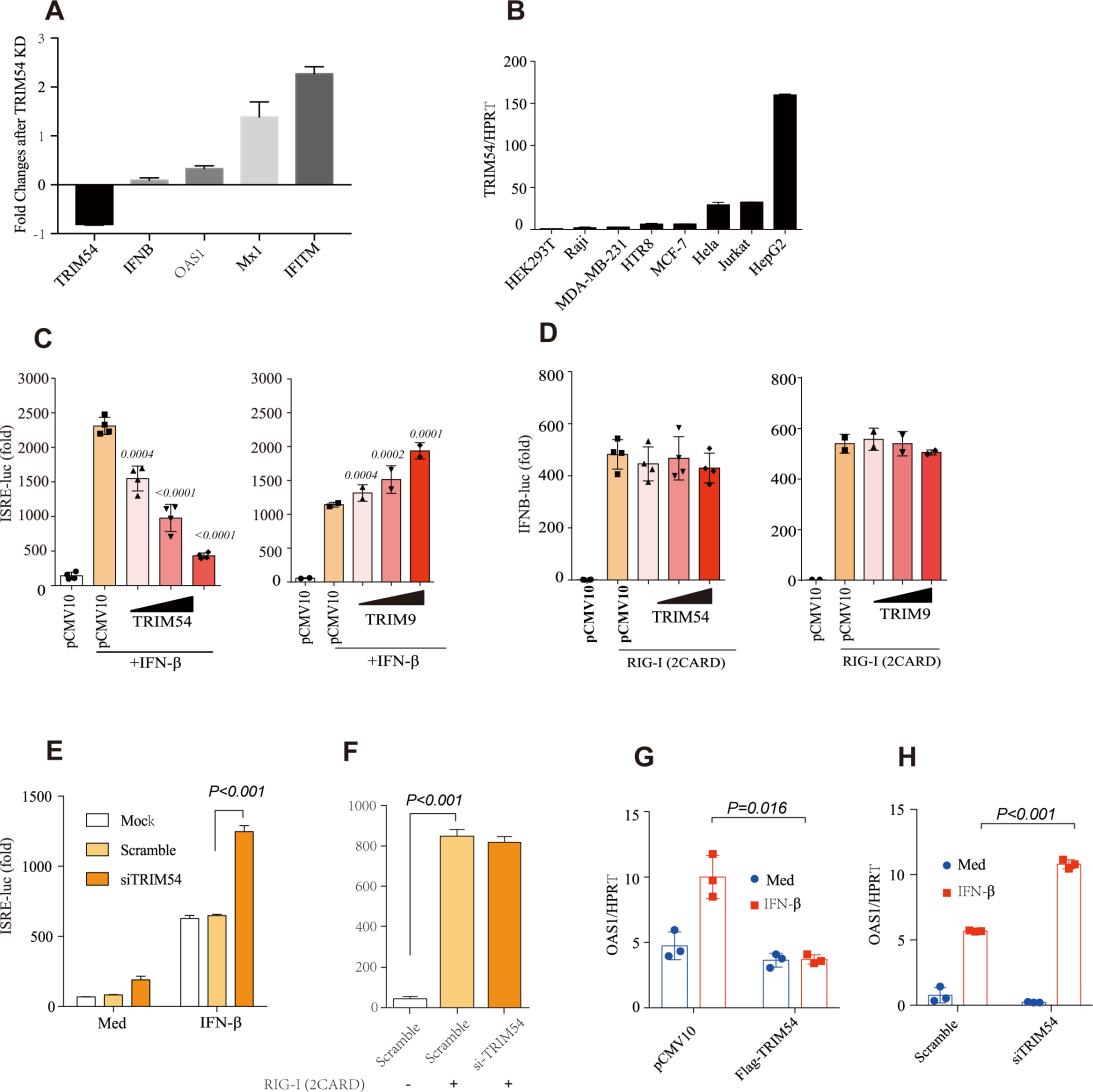
TRIM54 downregulates IFN-β signaling. (**A**) Human monocytes that had been transfected with either scrambled RNA (scRNA) or TRIM54-specific siRNA (siTRIM54) were stimulated with cIgG in triplicate wells for 24 h and then subjected to Q-PCR detection of TRIM54, IFNB, and ISG (OAS1, Mx1, IFITM) expression with HPRT as internal control. The results are expressed as *fold change* (triplicate well mean ± SD) of gene expression levels comparing the scRNA and siTRIM54 groups, calculated using the formula (siTRIM54 − scRNA)/scRNA. (**B**) Q-PCR screening for TRIM54 expression in human tumor cell lines, including HEK293T, Raji, MDA-MB-231, HTR8, MCF-7, Hela, Jurkat, and HepG2. The results are expressed as mean TRIM54/HPRT ± SD of three independent experiments. (**C, D**) HEK293T cells harboring luciferase reporters for ISRE or IFNB promoters were transfected with pCMV10 (300 nM) or pCMV10-based constructs encoding TRIM54 (left panels) or TRIM9 (right panels), at 30, 100, or 300 nM concentrations as indicated by the triangular symbols. These transfectant cells were then stimulated for 12 h with IFN-β (**C**), or RIG-I (2CARD) (**D**), followed by luciferase activity measurement. Unstimulated pCMV10-transfectant cells were included as additional controls. (**E, F**) HepG2 cells harboring luciferase reporters for ISRE or IFNB promoters were transfected with either scrambled RNA (scRNA) or siTRIM54, and then stimulated for 12 h with IFN-β (**E**) or RIG-I (2CARD) (**F**), followed by luciferase activity measurement. Mock transfection cells (Mock) were included as additional controls. The results are expressed as mean luciferase activity ± SD. (**G**) HEK293T cells harboring pCMV10 or pCMV10-Flag-TRIM54 plasmid were stimulated for 12 h with or without (Med) IFN-β (10 ng/mL). (**H**) HepG2 cells that had been transfected with scrambled RNA (Scramble) or TRIM54-specific siRNA (siTRIM54) were stimulated for 12 h with or without (Med) IFN-β (10 ng/mL). (**G, H**) OAS1 expression was determined by Q-PCR using HPRT as internal control, and the results expressed as mean OAS1/HPRT ± SD. The data shown are representative of at least three independent repeating experiments.

The correlation between induced TRIM54 expression and modulated IFN-I responsiveness (upregulation of IFN-β production and downregulation of ISG activation) in cIgG-treated human monocytes suggests a possibility that TRIM54 could regulate IFN-I production and/or signaling. Indeed, siRNA-mediated knockdown of TRIM54 in Mo(cIgG) cells significantly enhanced DENV-induced expression of ISGs (*OAS1, Mx1, IFITM*) without affecting IFNB transcription (Fig. 3A), indicating that TRIM54 selectively attenuates IFN-I signaling rather than IFN-I production.

Next, we employed human tumor cell lines for further assessment on the regulatory effects of TRIM54 on IFN-I responsiveness. Of the eight tumor cell lines PCR-screened for TRIM54 expression, HEK293T and HepG2 were chosen as representative low (TRIM54^lo^) and high (TRIM54^hi^) expresser lines, respectively (Fig. 3B). In the TRIM54^lo^ HEK293T cells, transfection of a human TRIM54-encoding DNA construct dose-dependently suppressed ISRE luciferase reporter gene expression elicited by IFN-β stimulation, while transfection of a TRIM9-encoding construct produced opposite effect (Fig. 3C). Meanwhile, transgenic overexpression of neither TRIM54 nor TRIM9 affected 2CARD (RIG-I agonist)-induced IFN-β-luciferase reporter gene expression in HEK293T cells (Fig. 3D). By contrast, siRNA-mediated TRIM54 knockdown in the TRIM54^hi^ HepG2 cells significantly augmented IFN-β- but not 2CARD-stimulated ISRE luciferase reporter gene expression (Fig. 3E and F). Moreover, *OAS1* transcription was suppressed by transgenic expression of Flag-TRIM54 in HEK293T cells but upregulated by siRNA-mediated TRIM54 knockdown in HepG2 cells (Fig. 3G and H). Thus, TRIM54 could function as an effective inhibitor of IFN-I signaling in human monocytic cells.

### STAT2 is selectively targeted by TRIM54

IFNAR2 is the major cell surface receptor for IFN-Is (1–3). We first examined whether TRIM54 influences IFN-I signaling by modulating IFNAR expression. Surface levels of IFNAR1 and IFNAR2 were comparable between Mo(cIgG) and Mo(RPMI) monocytes (Fig. 1A; Fig. S1B). Moreover, overexpression of TRIM54 in HEK293T cells did not alter IFNAR1 or IFNAR2 surface expression (Fig. S1C and D). These data argue against the possibility that TRIM54 suppresses IFN-I signaling through downregulation of IFNARs.

**Fig 4:**
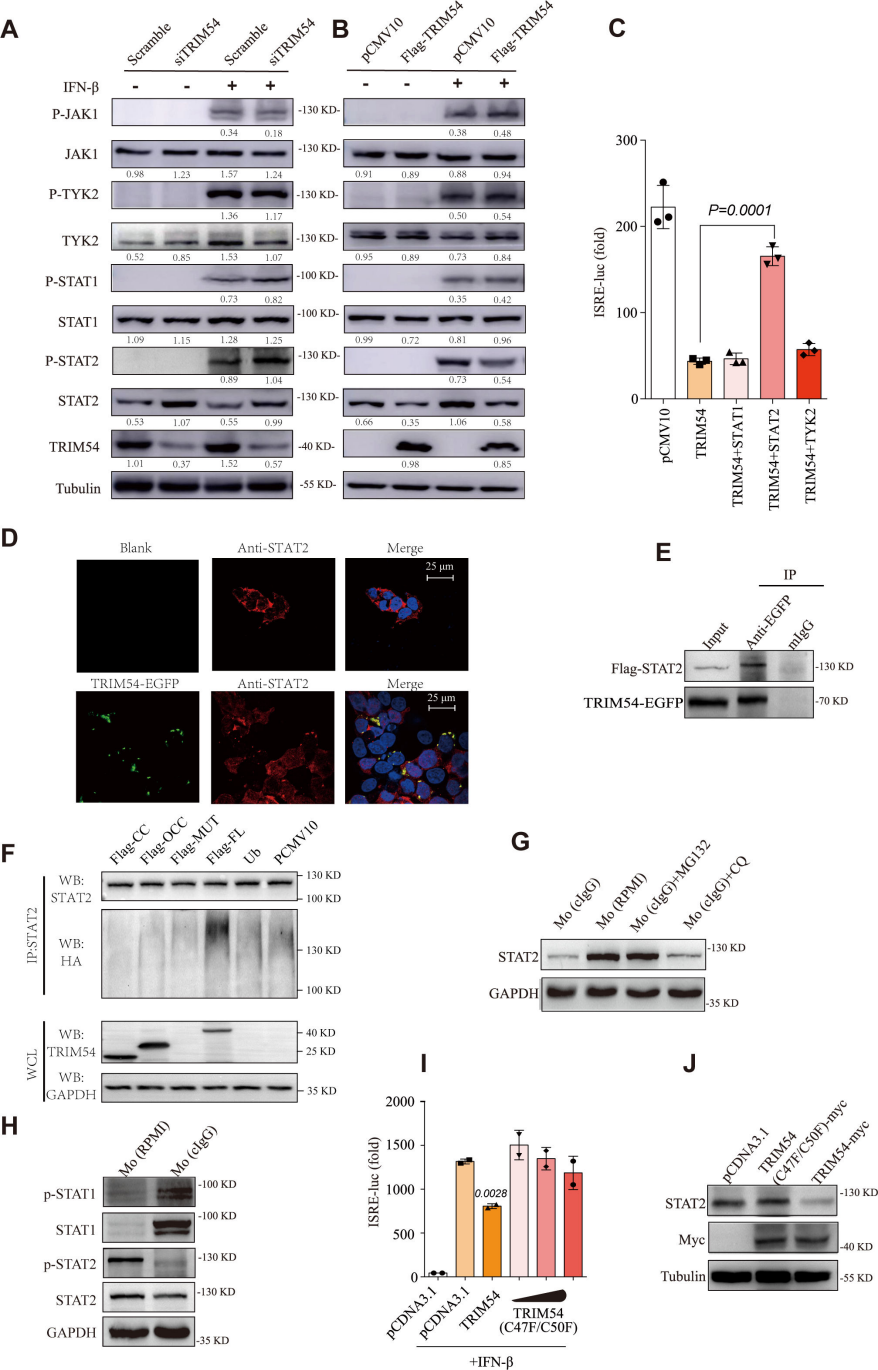
STAT2 is targeted by TRIM54. HepG2 cells transfected with either scramble or siTRIM54 RNA (**A**), and HEK293T cells harboring pCMV10 or pCMV10-Flag-TRIM54 plasmid (**B**) were stimulated for 10 mins with (+) or without (−) IFN-β (10 ng/mL), and then subjected to cell lysis and immunoblotting using Abs against JAK1, phosphorylated JAK1 (P-JAK1), TYK2, phosphorylated TYK2 (P-TYK2), STAT1, phosphorylated STAT1 (P-STAT1), STAT2, phosphorylated STAT2 (P-STAT2), and TRIM54 as probes. An Ab against tubulin was also included as a control. The semi-quantitation of WB was shown below each band. (**C**) HEK293T cells harboring luciferase reporter for the ISRE promoter were transfected with pCMV10-Flag-TRIM54 either alone (TRIM54), or together with DNA construct encoding STAT1 (TRIM54 + STAT1), or STAT2 (TRIM54 + STAT2), or TYK2 (TRIM54 + TYK2). Cells transfected with pCMV10 alone were included as an additional control. After 12 h IFN-β (10 ng/mL) stimulation, the transfectant cells were subjected to luciferase activity measurement, and the results expressed as mean luciferase activity ± SD. (**D**) HEK293T cells expressing TRIM54-EGFP were intracellularly stained with PE-labeled Ab against STAT2 in the presence of 10 μM/mL MG132 (proteasomal inhibitor). Cell nuclei were stained with DAPI (blue). Fluorescent images were captured with Nikon A1 confocal microscope through green (TRIM54-EGFP) and red (Anti-STAT2) channels. Merged micrograph shows intracellular co-localization of TRIM54 and STAT2. (**E**) Cell lysate from transfectant HEK293T cells co-expressing Flag-STAT2 and EGFP-TRIM54 was subjected to immunoprecipitation (IP) using either irrelevant mouse IgG (mIgG) or anti-EGFP IgG. The resultant precipitates were loaded onto SDS-PAGE 10% gel for WB using Abs against STAT2 (upper blot) or EGFP (lower blot) as probes. Unprocessed cell lysate (Input) was loaded as an additional control. (**F**) Lysates of HEK293T cells that had been transfected with pCMV10 or pCMV10-Flag-TRIM54, and other mutant fragment plasmids were precipitated using anti-STAT2 Ab. The resultant precipitates were loaded onto SDS-PAGE 10% gel for WB using HRP-conjugated Abs against STAT2 (upper blot) or hemagglutinin (HA) tag (lower blot) as probes. (**G**) Human monocytes were primed with [Mo(cIgG)] or without [Mo(RPMI)] cIgG in the presence or absence of MG132 (10 μM/mL) or CQ (5 μM/mL) for 24 h, followed by cell lysis and then WB using HRP-conjugated Abs against STAT2 (upper blots) and GAPDH (lower blots). (**H**) Mo(cIgG) and Mo(RPMI) cells that had been treated with IFN-β (10 ng/mL) for 30 min were lysed for WB using HRP-conjugated Abs against STAT1, p-STAT1, STAT2, p-STAT2, or GAPDH. (**I**) HEK293T cells that had been transiently transfected with pcDNA3.1 (10 nM), or pcDNA3.1-TRIM54 (10 nM), or pcDNA3.1-TRIM54(C47F/C50F) at increasing concentrations of 10, 30, and 100 nM as indicated by the triangular shaped symbol were stimulated with IFN-β for 12 h, followed by measurement of ISRE-luciferase activity. The results are expressed as mean luciferase activity ± SD. (**J**) Lysates of HEK293T cells that had been transfected with pcDNA3.1 or pcDNA3.1-based construct encoding Myc-tagged TRIM54 or TRIM54 (C47F/C50F) mutant were run on SDS-PAGE 10% gel for WB using HRP-conjugated Abs against STAT2 (upper blot), Myc (middle blot), or tubulin (lower blot). The data shown are representative of at least three independent repeating experiments.

We next investigated key signaling molecules downstream of IFNAR2, namely JAK1, TYK2, STAT1, and STAT2 (3). Knockdown of TRIM54 in IFN-β-stimulated or unstimulated TRIM54^hi^ HepG2 cells resulted in intracellular accumulation of STAT2, but not JAK1, TYK2, or STAT1 (Fig. 4A). Conversely, overexpression of TRIM54 in TRIM54^lo^ HEK293T cells reduced intracellular STAT2 levels without affecting JAK1, TYK2, or STAT1 (Fig. 4B). The inverse correlation between TRIM54 expression and STAT2 abundance suggested that STAT2 is a likely target of TRIM54. Several lines of evidence support this hypothesis: (i) the impaired IFN-β response in TRIM54-overexpressing HEK293T cells was rescued by co-transfection of STAT2 but not by STAT1 or TYK2 (Fig. 4C); (ii) confocal microscopic observation of TRIM54-STAT2 co-localization in EGFP-TRIM54-expressing HEK293T cells (Fig. 4D); (iii) TRIM54-STAT2 co-precipitation from lysate of HEK293T cells expressing EGFP-TRIM54 and Flag-STAT2 (Fig. 4E).

The antiviral or immunoregulatory activities of TRIMs rely, for the most part, on their function as E3-ubiquitin ligase, which is critically dependent on a conserved “C-X-X-C” motif (cysteine residues at positions 47 and 50) in the RING domain, evidenced by loss of function for TRIM54 mutant (TRIM54-C47F/C50F) carrying C47F and C50F point mutations (9–11). Consistent with the essential role of the RING domain E3 ligase activity, the TRIM54-C47F/C50F mutant neither suppressed IFN-β-induced ISRE-luciferase reporter activity nor promoted STAT2 degradation in transfected HEK293T cells (Fig. 4I and J). Moreover, HA-specific Western blotting assays confirmed ubiquitination of the STAT2 protein precipitated from lysate of transfectant HEK293T cells overexpressing wild type (WT) TRIM54 but not the RING and B-BOX-deletion (OCC) or the TRIM54-C47F/C50F mutant (Fig. 4F). It is of interest to note that STAT2 degradation in Mo(cIgG) cells was inhibitable by proteasome inhibitor MG132 but not by lysosome inhibitor chloroquine (Fig. 4G), and also that accelerated degradation of STAT2 and phosphorylated STAT2 (p-STAT2) in these monocytes was accompanied by marked accumulation of STAT1 and p-STAT1 (Fig. 4H). Together, these data indicate that TRIM54 selectively targets STAT2 for ubiquitination and subsequent proteasomal degradation, thereby attenuating IFN-I signaling.

### Structural characterization of TRIM54-STAT2 interaction

**Fig 5:**
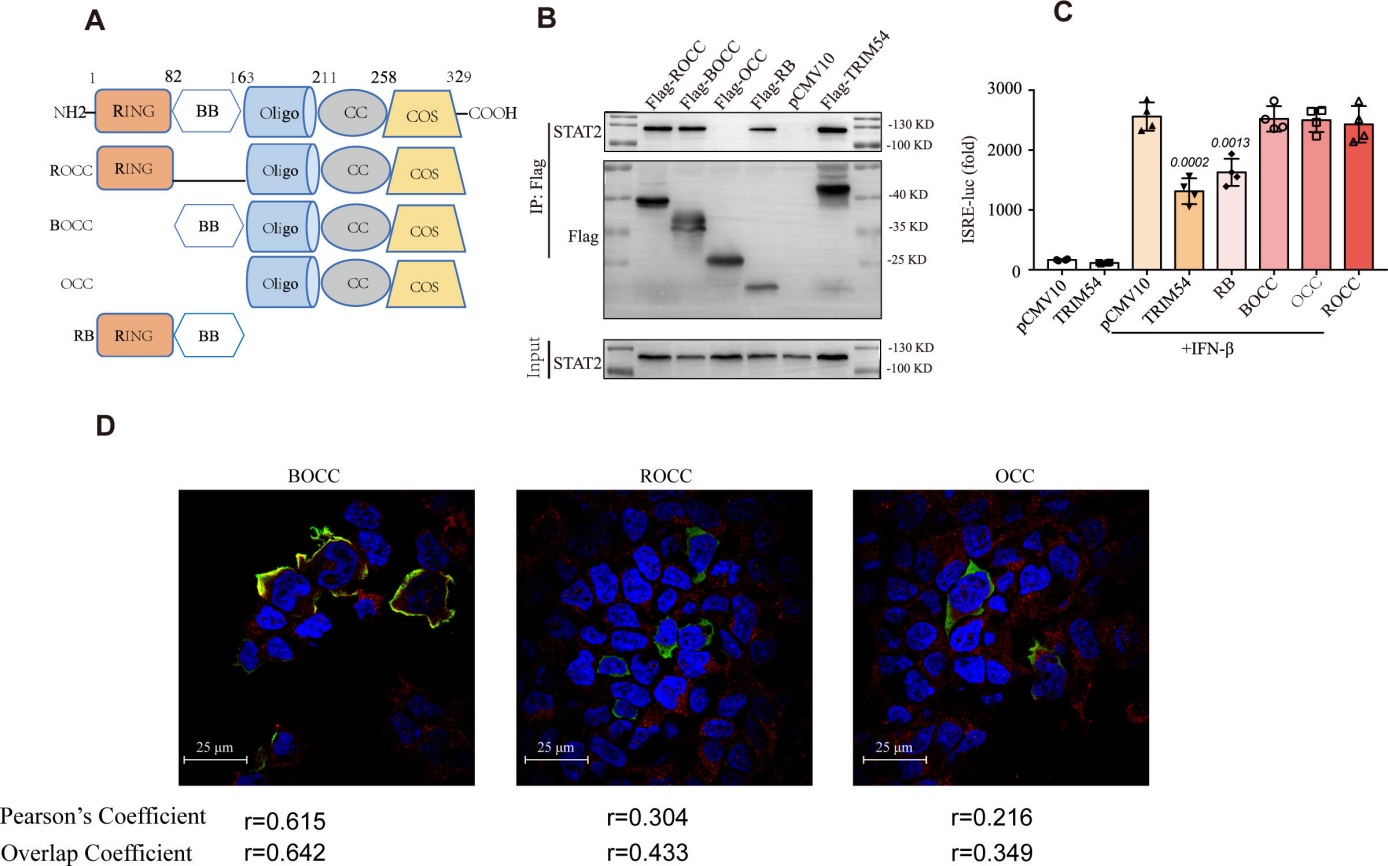
Structure-function relationship of TRIM54. (**A**) Schematic diagram showing the domain structures of TRIM54 and recombinant Flag-tagged TRIM54 deletion mutants ROCC, BOCC, OCC, or RB utilized in this study. (**B**) HEK293T cells were transiently transfected with either pCMV10 or pCMV10-based constructs encoding Flag-TRIM54, Flag-ROCC, Flag-BOCC, Flag-OCC, or Flag-RB, followed by cell lysis and then immunoprecipitation using anti-Flag Ab-coupled beads. The resultant precipitates were run on SDS-PAGE 10% gel for WB using HRP-conjugated Abs against STAT2 (upper blot) or Flag (lower blot) as probes. (**C**) HEK293T cells harboring luciferase reporter for the ISRE promoter were transfected with pCMV10 or pCMV10-based constructs encoding TRIM54 or its deletion mutants RB, BOCC, OCC, or ROCC. After 12 h IFN-β (10 ng/mL) stimulation, the transfectant cells were subjected to luciferase activity measurement, and the results expressed as mean luciferase activity ± SD. Unstimulated cells harboring pCMV10 or pCMV10-TRIM54 plasmid were also included as control. (**D**) HEK293T cells expressing EGFP-fused BOCC, ROCC or OCC mutants were intracellularly stained with PE-labeled Ab against STAT2 in the presence of MG132. Cell nuclei were stained with DAPI (blue). Fluorescent images were captured with Nikon A1 confocal microscope through green (EGFP-TRIM54 mutants) and red (PE-labeled anti-STAT2) channels. Merged micrographs are presented. Pearson’s coefficient and overlapping coefficient for the green and red staining are indicated at the bottom. The data shown are representatives of three independent repeating experiments.

TRIM superfamily proteins are characterized by a conserved domain structure including a N-terminal RING domain, one or two B-box motif(s), a coiled-coil region, and a C-terminal COS domain (16, 17). To define the structural determinants governing TRIM54–STAT2 interaction, we generated a series of Flag-tagged TRIM54 deletion mutants lacking the RING domain (BOCC), the B-box (ROCC), both the RING and B-box (OCC), or the coiled-coil region plus the COS domain (RB) (Fig. 5A). These constructs were transfected into TRIM54^lo^ HEK293T cells, followed by co- immunoprecipitation (Co-IP) assay using anti-TRIM54 Abs. Interestingly, deletion of either the RING domain (BOCC) or the B-box (ROCC) alone did not abolish the association between TRIM54 and STAT2. In contrast, the double-deletion mutant OCC (lacking both RING and B-box) failed to co-precipitate STAT2 (Fig. 5B). Consistent with the binding data, overexpression of the OCC mutant did not reduce STAT2 protein levels in whole-cell lysates (“*Input*” in Fig. 5B). These observations suggest that either the RING or the B-box domain is sufficient to mediate STAT2 binding with appreciable affinity. Functional analysis, however, revealed that neither domain was dispensable for TRIM54-mediated suppression of IFN-β-induced ISRE-luciferase activity: both the BOCC and ROCC mutants lost the ability to inhibit reporter gene expression (Fig. 5C). This suggests that while each domain can independently bind STAT2, both are required for the downstream inhibitory function—possibly because B-box-mediated tethering of STAT2 is a prerequisite for RING-dependent ubiquitination. We cannot exclude the possibility that an additional adaptor protein bridges TRIM54 to STAT2 in the functional complex. Importantly, deletion of the oligomerization/coiled-coil and COS domains (RB mutant) did not affect STAT2 binding or inhibition of IFN-β signaling (Fig. 5B and C), indicating that these regions are not essential for the interaction or function described here. To further corroborate the binding data, we prepared DNA construct-encoding EGFP-fused RB, BOCC, ROCC, or OCC mutants that were transfected into HEK293T cells. The transfectant HEK293T cells, after staining with PE-labeled STAT2 Ab, were then subjected to confocal microscopic examination. Consistent with the Co-IP results, EGFP-tagged BOCC and ROCC—but not OCC—showed clear co-localization with STAT2 (Fig. 5D), albeit the EGFP-RB group did not give meaningful results due to poor expression of the construct.

### FcγRIIa-TRIM54-STAT2 axis controls virus permissiveness of monocytic cells

**Fig 6:**
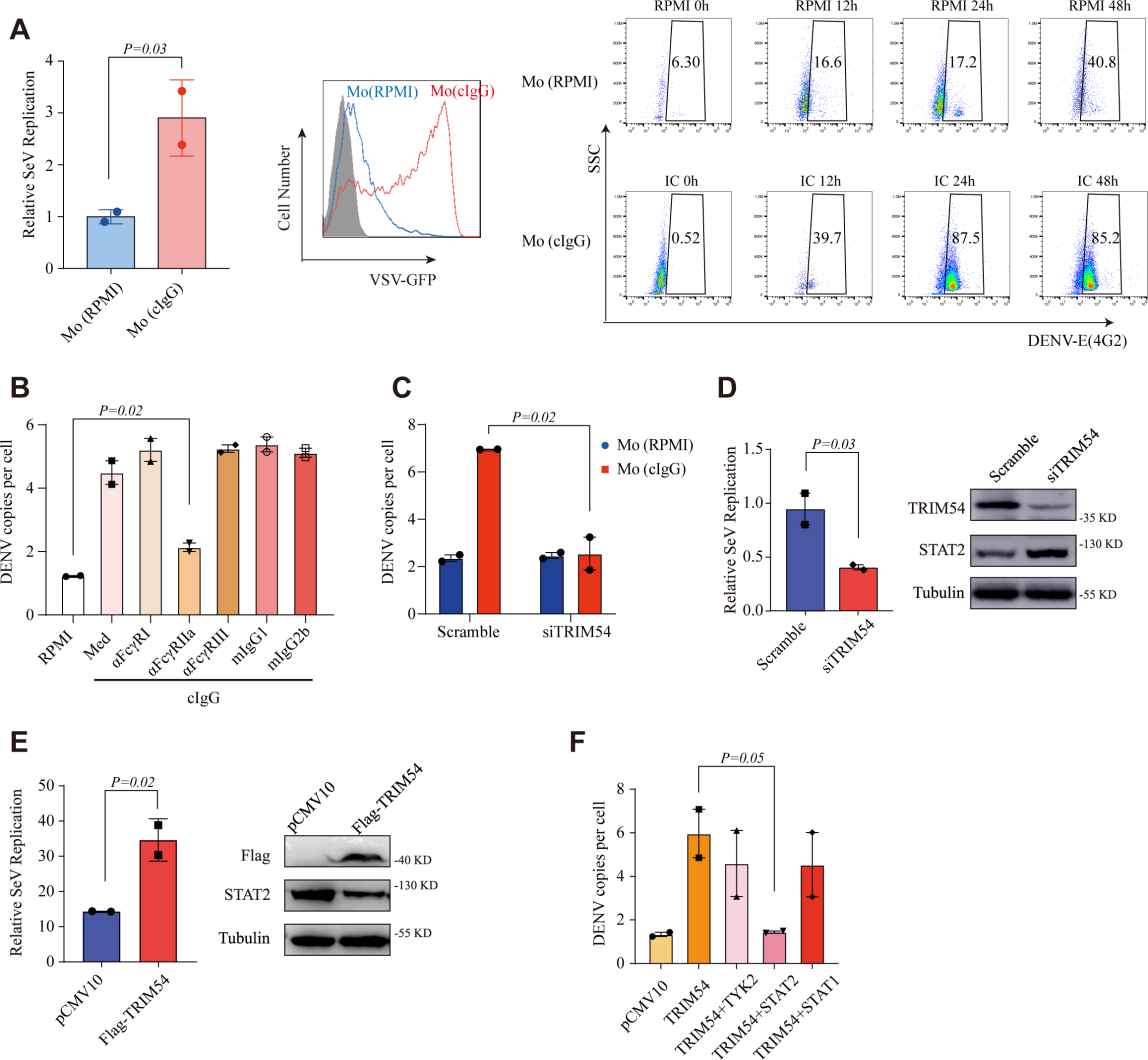
FcγRIIa-TRIM54-STAT2 axis regulates virus permissiveness. (**A**) Mo(RPMI) and Mo(cIgG) cells were infected for 12 h with either SeV (left panel) or GFP-expressing VSV (VSV-GFP) (middle panel), followed by assessment of relative viral replication using SeV HN-specific Q-PCR or flow cytometric analysis of GFP fluorescence intensity. Mo(RPMI) and Mo(cIgG) cells were infected with DENV-2 (right panels) for 0, 12, 24, and 48 h and then subjected to cytometric analysis of DENV-E (4G2) protein expression. (**B**) Fractionated blood monocytes were cultured for 24 h in human IgG-coated wells in the presence or absence (*Med*) of blocking Abs against FcγRI, FcγRIIa or FcγRIII (10 μg/mL), or isotype control mouse Abs (mIgG1 and mIgG2b), and then infected with DENV for 12 h, followed by Q-PCR detection of DENV E gene transcription. Monocytes cultured in uncoated wells alone (*RPMI*) were also infected with DENV as additional control. The results are expressed as RNA copies of DENV per cell. (**C**) Human blood monocytes, transfected with either scrambled RNA (*Scramble*) or TRIM54-specific siRNA (*siTRIM54*) by electroporation, were primed with [*Mo(cIgG*)] or without [*Mo(RPMI*)] cIgG, followed by 12 h DENV infection. Q-PCR was carried out for detection of DENV E gene transcription in the infected cells, and the results (mean ± SD) are expressed as RNA copies of DENV per cell. (**D**) HepG2 cells were pretreated with either scrambled RNA (*Scramble*) or TRIM54-specific siRNA (*siTRIM54*) by electroporation, followed by 24-h infection with SeV. Viral replication was determined using SeV HN-specific Q-PCR (left panel). The infected cells were also subjected to WB using Abs against TRIM54, STAT2, or tubulin as probes (right panel). (**E**) HEK293T cells transiently transfected with pCMV10 or pCMV10-Flag-TRIM54,were infected for 24 h with SeV, followed by Q-PCR detection of SeV HN gene transcription (left panel). The results (mean ± SD) are expressed as relative viral replication using the readout of the pCMV10 group as reference. WB was performed on the infected cells using Abs against Flag, STAT2, or tubulin as probes (right panel). (**F**) HEK293T cells were transiently transfected with either pCMV10-Flag-TRIM54 alone (*TRIM54*) or together with pCMV10 encoding STAT1 (*TRIM54 + STAT1*), or STAT2 (*TRIM54 + STAT2*), or TYK2 (*TRIM54 + TYK2*), followed by 24-h DENV infection. HEK293T cells transfected with pCMV10 were included as control. Q-PCR was carried out for detection of DENV E gene transcription, and the results (mean ± SD) are expressed as RNA copies of DENV per cell. The data shown are representative of at least three independent repeating experiments.

Because TRIM54 inhibits IFN-I signaling, we hypothesized that its induced expression in monocytes would enhance cellular permissiveness to viral infection. Consistent with this, Mo(cIgG) cells supported significantly higher levels of viral replication than Mo(RPMI) cells, as shown by elevated SeV gene transcription (Q-PCR) and increased VSV-EGFP and DENV-E protein expression (flow cytometry) (Fig. 6A). The ability of cIgG to induce viral permissiveness in monocytes was blocked by mAb against hFcγRII but not by Abs specific for hFcγRI or hFcγRIII (Fig. 6B). Additionally, siRNA-mediated TRIM54 knockdown in Mo(cIgG) cells led to significant reduction of DENV replication compared with the control cells (Fig. 6C), further supporting the role of TRIM54 in augmenting viral susceptibility. In TRIM54^hi^ HepG2 cells, siRNA-mediated TRIM54 knockdown significantly reduced intracellular SeV replication while increasing STAT2 accumulation (Fig. 6D). Conversely, overexpression of TRIM54 in TRIM54^lo^ HEK293T cells promoted viral permissiveness, an effect that was rescued by co-expression of STAT2 but not of STAT1 or TYK2 (Fig. 6E and F). Collectively, these results demonstrate that the FcγRIIa–TRIM54–STAT2 axis serves as a key regulatory circuit controlling viral permissiveness in human monocytic cells.

## DISCUSSION

Our findings reveal a novel mechanism through which a key component of adaptive immunity—virus-specific Abs—can, under certain conditions, such as at sub-neutralizing concentrations, suppress the IFN-I signaling cascade via a defined receptor-E3 ligase-transcription factor axis. In this pathway, IC-induced TRIM54 promotes STAT2 ubiquitination and degradation, thereby inhibiting ISG activation and enhancing cellular susceptibility to viral infection. The study draws on multiple viral models, including negative-sense (VSV and SeV) and positive-sense (DENV) single-stranded RNA viruses, and is further supported by clinical evidence from a DNA virus infection (EBV). We propose that the hFcγRIIa–TRIM54–STAT2 inhibitory axis could be exploited by diverse viruses, irrespective of their genome composition or replication strategy, to subvert cell-intrinsic antiviral defenses through ADE. Future studies should investigate whether therapeutic modulation of this axis can ameliorate severe disease outcomes linked to enhancing Abs.

ADE has been documented in infections caused by a range of viruses, such as DENV, Ebola virus, hepatitis C virus, coronavirus, and Ross River virus (12, 13, 26–30). Our work emphasizes that, for viruses capable of forming infectious ICs, the ADE phenomenon may not only increase cellular entry via FcγRs but also actively suppress the IFN-I-mediated antiviral state intracellularly. Extrinsic ADE refers to the increase in the number of cells infected via FcγR-mediated entry of virus-Ab complex, whereas intrinsic ADE results from enhanced viral production per cell due to suppression of IFN-I signaling and/or induction of IL-10 (31, 32). Our model posits that IgG-virus complexes cross-link hFcγRIIa to induce TRIM54 expression in monocytes, which in turn promotes STAT2 degradation and blunts IFN-driven ISG activation, thereby rendering cells more permissive to infection. In this setting, IC-induced TRIM54 acts as a “guilty player” in intrinsic ADE. This adds a novel signaling-specific dimension to intrinsic ADE, moving beyond entry facilitation. It should also be mentioned that, although hFcγRIIa is an important player in ADE (12, 13, 27, 33), mechanistic studies also indicate roles for FcγRIa and FcγRIIIa in promoting ADE during DENV infection (34, 35). It will be of interest to address the question if crosslinking of these FcγRs can also lead to dysregulation of cellular antiviral state.

While many TRIM proteins regulate IFN-I‐mediated antiviral and antitumor response (4), TRIM54 is, to our knowledge, the first TRIM family member shown to target STAT2 for intracellular signaling modulation. This finding is notable given the scarcity of known endogenous regulators of STAT2. Conversely, promoting STAT2 degradation is a common viral strategy to antagonize IFN responses (36). For example, Zika virus binds and degrades STAT2 in a proteasome-dependent manner (37, 38); herpes simplex virus type-2 (HSV-2) can cause selective loss of STAT2 transcript and protein or inhibit its phosphorylation and nuclear translocation (39); the NS2 protein of respiratory syncytial virus (RSV) induces STAT2 degradation in respiratory epithelium (40); and the DENV NS5 protein recruits the host E3 ligase UBR4 to ubiquitinate and degrade STAT2 (41).

Although endogenous TRIM54-STAT2 complex formation in primary monocytes after cIgG treatment was difficult to visualize by immunofluorescence and confocal microscopy, we observed a clear correlation between induced TRIM54 expression and accelerated proteasomal degradation of STAT2 (Fig. 4G and H). Moreover, mass spectrometry analysis of STAT2 immunoprecipitated from Mo(cIgG) cells confirmed its ubiquitination (Fig. S2).

An unexpected observation in this study is the dual (“Janus”) effect of IgG ICs on IFN-I responsiveness in human monocytes: (i) sensitization for enhanced IFN-I production upon PAMP or viral stimulation (Fig. 1A and B), and (ii) suppression of IFN-I signaling through TRIM54 induction (Fig. 2 and 3). The former effect is possibly linked to marked STAT1 accumulation in cIgG-treated monocytes (Fig. 4H). This “Side A” phenomenon aligns with an earlier report that FcγRIIa on plasmacytoid dendritic cells is required for IFN-α production triggered by apoptotic cells combined with lupus IgG (42) but contrasts with a recent study showing that FcγRIIa suppresses IFN-I and IFN-III production in human myeloid cells (43)—a discrepancy possibly attributable to differences in cell types and experimental stimuli. The “Side B” effect is consistent with earlier work demonstrating that ICs can suppress IFN-γ-induced responses in monocytes via activation of specific SRC-family kinases (44, 45). It is worth noting that certain antiviral ISGs (e.g., RSAD2) were not suppressed by the “Side B” mechanism (Fig. 1E). Given that TRIM54 overexpression did not alter RIG-I-driven activation of IFN-I promoters (Fig. 3), genes that are directly inducible by IRF3 (e.g., RSAD2) may operate outside the TRIM54-STAT2 axis. Alternatively, there is substantial evidence that STAT2 forms a variety of transcription factor complexes (46, 47), and differences in the architecture of STAT2-driven promoters among ISGs could also account for this selectivity.

ISG15, which was suppressed by cIgG pretreatment in human monocytes (Fig. 1E; Fig. S1C), has recently been identified as part of an ISG15/USP18/STAT2 hub that regulates IFN-I-mediated control of DENV and Zika virus replication (48, 49). It will be interesting to explore how the FcγRIIa/TRIM54/STAT2 and ISG15/USP18/STAT2 axes interact during viral infection in the presence of virus-Ab ICs.

We also found that human neutrophils—which express all three activating FcγRs—upregulate TRIM54 transcription after cIgG stimulation (Fig. S3A). Moreover, monocyte-derived DCs generated *in vitro* with GM-CSF showed strong TRIM54 positivity (Fig. S3B). These results suggest that, besides monocytes, other human myeloid cell types can be induced to express TRIM54 under appropriate stimulation. Future work should also examine FcγR-independent conditions that might trigger TRIM54 induction in non-immune cells, such as epithelial cells exposed to combined viral and inflammatory cytokine stimulation. Hyperinflammation is a common feature of viral infections, including dengue (50–52), and our data show that genes encoding cytokines and regulatory molecules are markedly upregulated in IC-conditioned monocytes under IFN stimulation (Fig. 2A and B). If tissue cells in a hyperinflammatory milieu express TRIM54, local susceptibility to viral infection could be increased.

In summary, the FcγRIIa–TRIM54–STAT2 axis represents a previously unrecognized pathway of cross-regulation between adaptive humoral immunity and innate IFN-I responsiveness—one that may be hijacked by viruses to facilitate intrinsic ADE. These insights advance our understanding of how Ab responses can inadvertently dampen antiviral defense and may inform the design of more effective vaccines against viral diseases.

## MATERIALS AND METHODS

### Abs, viruses, and reagents

Human IVIG was from Sinopharm. Mouse monoclonal Abs (mAbs) against human FcγRI (CD64, 10.1) or FcγRIII (CD16, 3G8), mouse IgG1 (ET901), and IgG2b (MPC-11) were from BioLegend. Mouse IgG2a (no. 16-4724-82) and mIgG3 (no. 14-4742-81) were from eBioscience. Anti-human FcγRIIa mAb (CD32a, IV.3) was from STEMCELL Technologies. PE-labeled anti-IFNAR2 or anti-IFNAR1 was from SinoBiological. Additionally, Abs against Flag, Myc, Histidine tag or human p-Jak1, Jak1, p-Tyk2, Tyk2, p-Stat1, Stat1, p-Stat2, Stat2, TRIM54, tubulin, or GAPDH was used for Western blotting (WB) experiments. More detailed information on Abs employed in this study is provided in Table S1.

GFP-expressing vesicular stomatitis virus (VSV-GFP), Sendai virus (SeV), and Dengue virus (DENV, DENV2) were generous gifts from Drs. CS Dong, FF Zhou and JF Dai of the Institute of Biology and Medical Sciences, Soochow University, China. DENV-2 NGC strain was propagated in mosquito C6/36 cells and titrated by plaque assay on Vero cells. Cells were infected with DENV, SeV, or VSV-GFP at a multiplicity of infection (MOI) of 1 for 1 h, unless otherwise stated, followed by removal of the inoculum, two washes with PBS, and the addition of fresh medium to ensure the measurement of *de novo* viral replication.

Recombinant human IFN-β was from PeproTech. Recombinant mouse IFN-β was from R&D. Inhibitors of signaling pathways, including Wortmannin, 3MA, LY294002, RAPA, AKTi (MK-2206 HCl), and R406, were purchased from Selleck (Shanghai, China). Poly I:C was from NOVUS.

### Plasmid construction

DNA fragments encoding full-length TRIM54 and TRIM54 domain deletions were PCR amplified from human monocytes primed with plate-bound IVIG (cIgG), and the resultant DNA cloned into vector p3 × FLAG-CMV-10 within Hind III and BamH I restriction sites using corresponding primers. Full-length TRIM54 was also cloned into expression vectors pcDNA3.1/myc-His and pEGFP-N1 within the same restriction sites. The MYC-tagged TRIM54 mutant C47A/C50A was created by site-directed mutagenesis using the FastPfu Fly DNA Polymerase (Transgene). The FLAG-tagged TRIM9 was constructed by cloning the full-length TRIM9 into vector p3 × FLAG-CMV-10 within *EcoR I* and *BamH I* restriction sites to fuse them in N-terminal with FLAG. Full-length STAT2 and STAT2 domain deletions were PCR amplified from human monocytes and cloned into pcDNA3.1/myc-His within *Kpn I* and *EcoR I* restriction sites with corresponding primers. Sequences of primers used for plasmid construction are given in Table S2.

### Human monocyte isolation and human tumor cell lines

Ficoll lymphocyte separating solution (Dakewe Biotech) was used to isolate peripheral blood mononuclear cells (PBMCs) from freshly drawn venous blood of healthy volunteers by density gradient centrifugation. Monocytes were then sorted from PBMCs using human CD14 magnetic labeled beads (MACS; Miltenyi Biotec) according to the manufacturer’s instructions. Purified monocytes (approximately 98% homogeneity as determined by FACS analysis) were cultured in “R10” medium: RPMI 1640 medium containing 10% autologous serum, 100 U/mL penicillin, and 100 µg/mL streptomycin at 37°C and 5% CO_2_.

Human tumor cell lines, including Raji, HEK293T, MDA-MB-231, HTR8, MCF-7, Hela, Jurkat, and HepG2, were purchased from American Type Culture Collection (ATCC). All tumor line cells were cultured at 37°C under 5% CO_2_ in DMEM medium supplemented with 10% Fetal Bovine Serum (FBS), 100 U/mL penicillin, and 100 µg/mL streptomycin.

### Blood samples from pediatric patients and control subjects

Specimen of peripheral blood from infectious mononucleosis (IM) patients (*n* = 13, age 3–6 years, admitted to Suzhou Children’s Hospital between 2019 and 2021) with >5 × 10^3^ EBV load were subjected to CD14^+^ monocyte isolation, followed by qRT-PCR detection of TRIM54 transcription. Blood samples from age- and gender-matched uninfected subjects (*n* = 21) were included as control. EBV load in blood was detected by real-time fluorescence quantitative PCR (EBV qPCR Kit, Sansure Biotech).

### DENV infection

Human monocytes (10^6^ cells) were seeded and infected with DENV. Virus was added to the cells in 200 μL per well. DENV copy numbers were determined by a standard curve generated from serial dilutions of a quantified standard using qRT-PCR CT values and the dilution factors.

For flow cytometric detection of DENV-infected cells, monocytes were harvested, fixed with 4% paraformaldehyde, and permeabilized with saponin. Cells were then stained with anti-DENV envelope monoclonal antibody 4G2 (Millipore), followed by donkey anti-mouse IgG conjugated to APC (Molecular Probes).

### RNA-seq

Total cellular RNA was extracted with the Total RNA Kit II (OMEGA). Library construction and sequencing were performed on a HiSeq or Novaseq 2 × 150 platform by AZENTA Life Sciences (Suzhou). Sequenced reads were mapped to reference human genome (hg19 assembly) using bowtie21 with default parameters, and Cufflinks^2^ was used to estimate the abundance of transcripts. Quantifications of gene expression were performed using RSEM, the expression levels of genes in each sample were normalized by means of fragments per kilobase of transcript per million mapped reads (FPKM).

### RNA-seq analysis

After eliminating absent features (zero counts), differential expressed genes (DEGs) between two groups with three replicates were compared via DEseq2, with the cutoff of fold change ≥1 and *P*-value < 0.05. These RNA-seq data were deposited in the GEO database with the accession number GSE 102728 and GSE190623. To find the signaling pathway enriched in differentially regulated genes, we used Gene Set Variation Analysis (GSVA) to calculate the significance score. Genes with significant change were visualized with heatmap, each column represents the mean expression of three replicates.

### RT-PCR and ELISA

Total cellular RNA was prepared using Omega RNA Isolation Kit and cDNA synthesis using First Strand cDNA Synthesis Kit (Takara). Quantitative RT-PCR (qRT-PCR) was performed on StepOnePlus Real-Time PCR System (Applied Biosystems) using SYBR Green Master Mix (Takara). mRNA levels were normalized to housekeeping gene expression. Primers used for RT-PCRs are listed in Table S3. ELISA kits for detection of IFN-β in culture supernatants were purchased from Fcmacs Biotech.

### Transfection and luciferase reporter assay

For TRIM54 overexpression, HEK293T cells were transfected with TRIM54-encoding expression vector using LongTrans *In Vitro* DNA Transfection Reagent (UCallM Biotech). For TRIM54 knockdown, HepG2 cells were transfected with TRIM54-specific siRNA, or scrambled sequence as control, using HiPerFect Transfection Reagent (QIAGEN). Purified monocytes (1 × 10^7^ cells/well) were transfected with siRNA using the Amaxa kit (Lonza, Basel, Switzerland) according to the manufacturer protocol. The cells were then plated on six-well plates and cultured for 6 h. The transfected cells were added to IgG-coated, or uncoated, plate wells, and incubated for 18–24 h.

For analysis of IFN-induced transcriptional activity, cells were transfected with the ISRE-Luciferase together with Renilla plasmids and pCMV10-TRIM54/TRIM9, or indicated TRIM54 fragment expression plasmid, or TRIM54 siRNA (siTRIM54). After 48 h, cells were treated with IFN-β for 24 h. For analysis of IFN-β production stimulated with SeV, HepG2 cells were transfected with IFN-β-luciferase and Renilla plasmids, together with siTRIM54. After 48 h, cells were infected with SeV for 16 h. For assays with stimulation of RIG-I(2CARD), HEK293T cells were co-transfected with IFN-β-luciferase, Renilla plasmids, RIG-I(2CARD), and pCMV10-TRIM54 or pCMV10-TRIM9. Luciferase activities were measured 48 h post-transfection with the Dual-Luciferase Assay (Promega) according to the manufacturer’s protocol. Results are shown as the ratios of firefly luciferase activity/Renilla luciferase activity.

### Western blotting

Cells were washed with ice-cold PBS and subsequently lysed with RIPA lysis buffer (1% NP-40, 0.5% deoxycholate, 0.1% SDS) containing protease inhibitors and phosphatase inhibitor cocktail (Selleck) for 10 min on ice. Protein concentration was measured by BCA protein assay kit (Thermo). Total cell lysate was separated by SDS-PAGE and transferred onto PVDF membranes. Immunoblots were blocked by 5% non-fat milk in PBST buffer for 1 h at room temperature and then incubated with primary antibodies against p-Jak1, Jak1, p-Tyk2, Tyk2, p-Stat1, Stat1, p-Stat2, Stat2, TRIM54, Flag, Myc, His, Tubulin, or GAPDH at 4°C overnight. The immunoreactive bands were detected by secondary anti-mouse or anti-rabbit antibodies conjugated to horseradish peroxidase (HRP) with ECL reagents.

### Co-immunoprecipitation

For Co-IP assays, HEK-293T cells in a six-well plate were co-transfected with the indicated expression plasmids or empty vectors. Cells were lysed in 300 μL lysis buffer (1% Triton X-100, Beyotime Biotech) plus protease inhibitors (Selleck) for 30 min on ice, and clarified by centrifugation at 12,000 × *g* for 15 min at 4°C. Briefly, the supernatant was incubated with mouse anti-EGFP (Proteintech, 50430-2-AP), anti-Flag (Proteintech, 20543-1-AP) or mouse IgG overnight on a rotor at 4°C. Then, 20 μL washed Protein A/G puls-agarose beads (Santa Cruze, sc-2003) was added, followed by an additional 6 h on a rotor at 4°C. Beads were washed five times with cold lysis buffer, and the immunoprecipitates were eluted with SDS loading buffer. Immunoblot analysis was performed as described above.

### Immunofluorescence staining, FACS and confocal microscopic analysis

HEK293T cells were transfected with TRIM54-ΕGFP. After 18 h, cells were then fixed with buffer containing citrate, acetone, and formaldehyde, and then blocked for 60 min with blocking solution (5%FBS, 0.3% Triton X-100), incubated with Rabbit anti-Stat2 monoclonal antibody (Cell Signaling, 72604) at 4°C overnight, followed by AlexaFluor-647-labeled secondary antibodies (eBioscience, San Diego, CA, USA). DAPI (Solarbio, Beijing, China) was used to visualize the nuclei. Images were captured with the Nikon (Tokyo, Japan) A1 laser scanning confocal microscope.

Monocytes or tumor line cells were stained with fluorescence-labeled Abs in PBS containing 1% BSA and 0.1% sodium azide. After wash-out, the fluorescence was assessed by flow cytometry (FACScan Canto II, BD Biosciences), and data were analyzed with Flowjo software (Flowjo, LLC).

### Statistical analysis

Two-tailed Student’s *t*-test was used to analyze the comparison between different groups. Data represent the mean ± sd. All differences were considered statistically significant when *P* < 0.05.

## Data Availability

RNA sequencing data used in this article have been deposited in the Gene Expression Omnibus database with accession number GSE 102728 and 190623.

## SUPPLEMENTAL MATERIAL

Supplemental material jvi.02106-25-s0001.docx Figures S1 to S3; Tables S1 to S3.

ASM does not own the copyrights to Supplemental Material that may be linked to, or accessed through, an article. The authors have granted ASM a non-exclusive, world-wide license to publish the Supplemental Material files. Please contact the corresponding author directly for reuse.
