# Supplementary material for: A novel FcγRIIa-TRIM54-STAT2 axis negatively regulates type I interferon signaling and promotes viral susceptibility in human monocytes
Source: J Virol. 2026 May 29;100(6):e02106-25. doi: 10.1128/jvi.02106-25 (PMC13289085; doi:10.1128/jvi.02106-25)
Supplement: Supplemental material — Figures S1 to S3; Tables S1 to S3. [file jvi.02106-25-s0001.docx]

**A Novel FcγRIIa-TRIM54-STAT2 Axis Negatively Regulates Type I Interferon Signaling and Promotes Viral Susceptibility in Human Monocytes**

Running title: TRIM54 in Type I IFN signaling regulation

Xiao-Qiu Dai^1,#^, Shenghao Hua^1, 2,#^, Lian Xue^1^, Zheng Gong^1^, Ya-Ying Pan^1^, Zhenjun Li^3^, Chaojie Han^1^, Xiao-Ming Gao^1,*^ and Fang-Yuan Gong^1,*^

^1^ School of Basic Medical Sciences, Soochow University, Suzhou, China;

^2^ Department of Clinical Laboratory, Children's Hospital of Soochow University, Suzhou, China

^3^ Department of Rheumatology, Kowloon Hospital, Suzhou, China.

*Correspondence: Drs. Fang-Yuan Gong or Xiao-Ming Gao, 199 Ren’ai Road, Suzhou 215123, China, Email: [gongfangyuan@suda.edu.cn](mailto:gongfangyuan@suda.edu.cn) or [xmgao@suda.edu.cn](mailto:xmgao@suda.edu.cn)

# These authors contribute equally to the work

**SUPPLEMENTAL MATERIALS**

Fig. S1 Flow cytometric analysis of IFNAR expression in monocytes treated with, or without, cIgG

Fig.S2 cIgG regulates STAT2 stability through ubiquitination in monocytes

Fig. S3 Induced TRIM54 expression in different types of myeloid cells

Table S1 Abs used in this study

Table S2 Plasmid construction primers

Table S3 Q-PCR primers


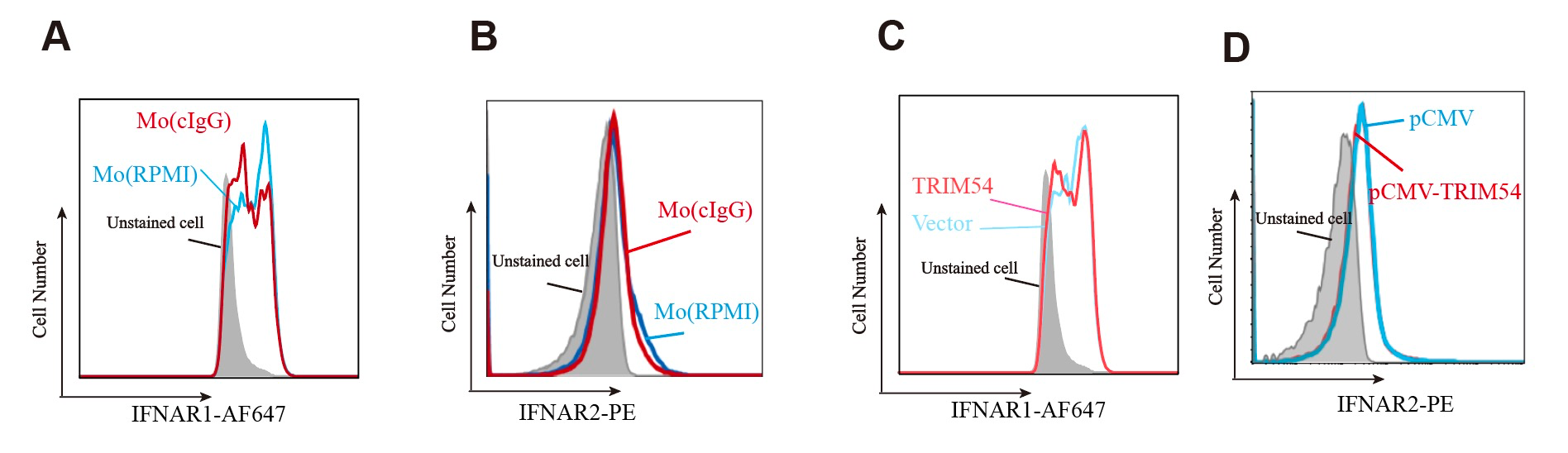
**Figure S1 Flow cytometric analysis of surface IFNAR1 and IFNAR2 expression in monocytes treated with, or witout, cIgG.** (**A**) Freshly isolated human monocytes were stimulated with [Mo(cIgG)], or without [Mo(RPMI)], cIgG for 24 h and then stained with PE-labeled anti-human IFNAR2 Ab, followed by FACS analysis. Representative histograms are shown, filled histograms represent unstained control cells. (**B**) HEK293T cells that had been transfected with pCMV10-Flag-TRIM54, or pCMV10, were stained with PE-labeled Ab against human IFNAR2, followed by FACS analysis.


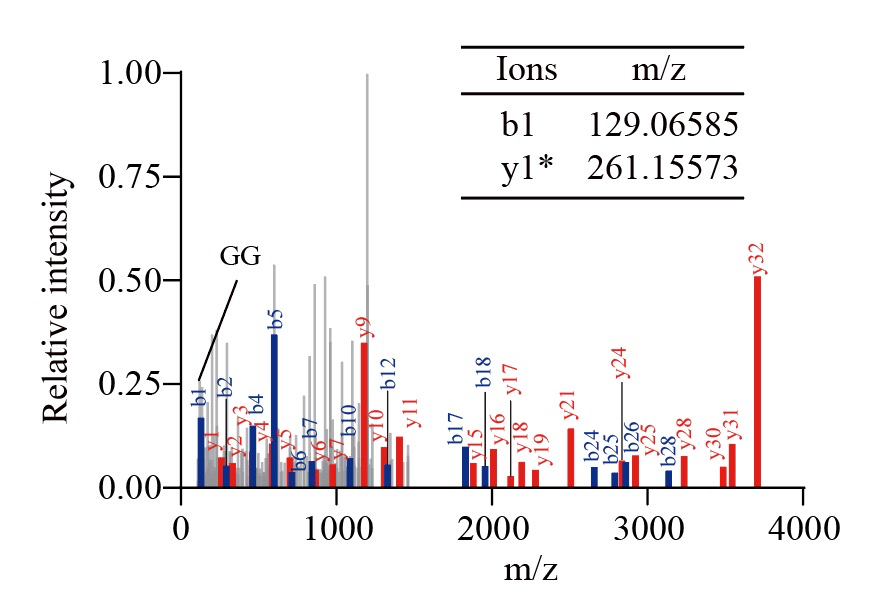


**Figure S2 cIgG regulate STAT2 stability through ubiquitination in monocytes**

Monocytes were treated with cIgG or med for 18 hours. Proteins were extracted and STAT2 was immunoprecipitated. The immunoprecipitated samples were analyzed by mass spectrometry to identify ubiquitination residues on the STAT2 protein.


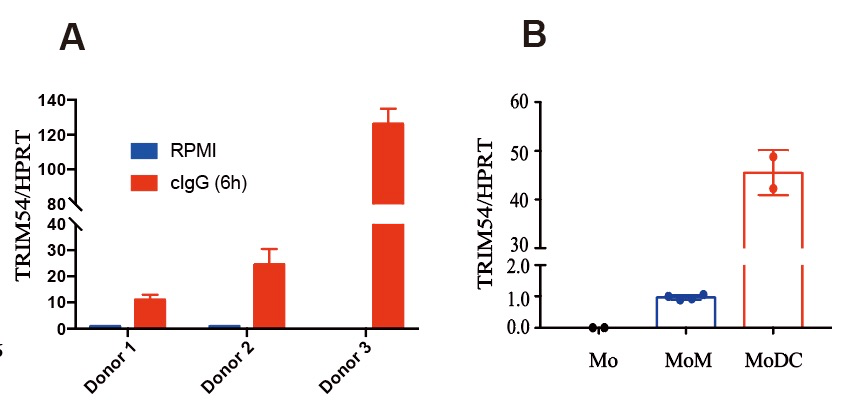


**Figure S3 Induced TRIM54 expression in different types of myeloid cells.** (**A**) Circulating neutrophils from 3 healthy blood donors were individually cultured for 6 h in the presence, or absence (RPMI), of cIgG. (**B**) Freshly isolated blood monocytes from healthy volunteers were differentiated into macrophages (moM) or dendritic cells (moDC) through 7 day M-CSF and GM-CSF stimulation, respectively. TRIM54 expression was assessed by Q-PCR using HPRT as internal control.

**Supplemental Table S1 Abs used in this study**

| **Name** | **Description** | **Clone** | **Sources** | |  |
| --- | --- | --- | --- | --- | --- |
| Anti-jak1 | Jak1 (6G4) Rabbit mAb | mAb | Cell Signaling | |  |
| Anti-p-jak1 | Phospho-Jak1 (Tyr1022) Rabbit mAb | Polyclonal Ab | | Santa Cruz |  |
| Anti-tyk2 | Human Tyk2 MAb (Clone 307214) | mAb | R&D | |  |
| Anti-p-tyk2 | Phospho-Tyk2 (Tyr1054/1055) (D7T8A) Rabbit mAb | mAb | Cell Signaling | |  |
| Anti-stat1 | Stat1 (D1K9Y) Rabbit mAb | Polyclonal Ab | Cell Signaling | |  |
| Anti-p-stat1 | Phospho-Stat1 (Tyr701) (D4A7) Rabbit mAb | Polyclonal Ab | Cell Signaling | | |
| Anti-stat2 | Stat2 (D9J7L) Rabbit mAb | mAb | Cell Signaling | | |
| Anti-p-stat2 | Phospho-Stat2 (Tyr690) (D3P2P) Rabbit mAb | mAb | Cell Signaling | |  |
| Anti-EGFP | EGFP tag Antibody | mAb | Proteintech | |  |
| Anti-Flag | DYKDDDDK Tag (D6W5B) Rabbit mAb | mAb | Cell Signaling | |  |
| Anti-Myc | Myc tag Antibody | mAb | Proteintech | |  |
| Anti-His | His tag Antibody | mAb | Proteintech | |  |
| Anti-TRIM54 | MURF3 Antibody | Polyclonal Ab | Invitrogen | |  |
| Anti-Tubulin | α/β-Tubulin Antibody | Polyclonal Ab | Cell Signaling | |  |
| Anti-GADPH | Anti-GAPDH antibody | Polyclonal Ab | Sigma-Aldrich | |  |
| PE-anti-IFNAR2 | anti-IFNAR2 Rabbit mAb | mAb | SinaBiological | |  |
| PE-anti-IFNAR1 | anti-IFNAR1 Rabbit mAb | mAb | SinaBiological | |  |
| Anti-Flavivirus envelope protein | Monoclonal Antibody (4G2) | mAb | NOVUS | |  |

**Supplemental Table S2 Plasmid construction primers**

| **Plasmid** | **Forward primer (5’-3’)** | **Reverse primer (5’-3’)** |
| --- | --- | --- |
| Flag -TRIM54 | CCAAGCTTATGAACTTCACAGTGGGTTT | CGGGATCCTTAAGGCCCATCCGGCCGCTCTT |
| Flag-TRIM9 | CGGAATTCATGGAGGAGATGGAAGAGGA | CGGGATCCTTAGATGCCACGCAGTTCTC |
| TRIM54-myc | CCAAGCTTATGAACTTCACAGTGGGTTT | CGGGATCCAGAGGCCCATCCGGCCGCTCTT |
| TRIM54-EGFP | CCAAGCTTATGAACTTCACAGTGGGTTT | CGGGATCCCGAGGCCCATCCGGCCGCTCTT |
| TRIM54-CFP | GCAAGCTTATGAACTTCACAGTGGGTTTC | GCGAATTCTTAAGGCCCATCCGGC |
| STAT2-YFP | CCGAATTCATGGCGCAGTGGGAAATGC | GCCTCGAGGAAGTCAGAAGGCATCAAG |
| CFP-YFP | GCGAATTCATGGTGAGCAAGGGCGAGG | GGCTCGAGTTACTTGTACAGCTCGTCC |
| ΔRING | ATAAGCTTGAGCAGCACCTCATGTGCGA | CGGGATCCAGGCCCATCCGGCCGCTCTT |
| ΔRBO | ATAAGCTTCTGTGCGCAGTGCTGGAGGA | CGGGATCCAGGCCCATCCGGCCGCTCTT |
| ΔRBOC | TCAAGCTTATGGAAGAGCCACAAATGGC | CGGGATCCAGGCCCATCCGGCCGCTCTT |
| ΔCOS | GGGAAGCTTATGAACTTCACAGTGGGTTT | TTTGGATCCGGACTGGATGGCAGACTCCA |
| ΔCC | ATAAGCTTTGCCCCATCTGCCTGGAGAT | TTGGATCCCTGCTTCTGCCTCCGGCTAT |
| ΔOCC | ATAAGCTTTGCCCCATCTGCCTGGAGAT | TTGGATCCCAGTGGGGCCACCTCACAGT |
| ΔBOCC | GGGAAGCTTATGAACTTCACAGTGGGTT | TTTGGATCCAGCCTTGGAGTGCAGCGGC |
| ΔBox F | CGCAAGCTTATGAACTTCACAGTGGGTTT | TGTAAATGGTGGGAGCCTTGGAGTGCAGCG |
| ΔBox R | GCACTCCAAGGCTCCCACCATTTACAAACG | ATTGGATCCAGGCCCATCCGGCCGCTCTT |
| ΔRB | TCAAGCTTAAACGCCAGAAGAGTGAGCT | CGGGATCCAGGCCCATCCGGCCGCTCTT |
| TRIM54(C24F) F | CGCAAGCTTATGAACTTCACAGTGGGTTT | CACATTTGCGGAACAGGTTGTGTTGGCAG |
| TRIM54(C24F) R | cacaacctgtTccgcaaatgtgccaacga | ATTGGATCCAGGCCCATCCGGCCGCTCTT |
| TRIM54 (C27F) F | CGCAAGCTTATGAACTTCACAGTGGGTTT | TCGTTGGCAAATTTGCGGAACAGGTTGTG |
| TRIM54 (C27F) R | gtTccgcaaatttgccaacgacgtcttcc | ATTGGATCCAGGCCCATCCGGCCGCTCTT |

**Supplemental Table S3 Q-PCR primers**

| **Gene** | | **Forward primer (5'-3')** | **Reverse primer (5'-3')** |
| --- | --- | --- | --- |
| IFN-β | | AGTAGGCGACACTGTTCGTG | AGCCTCCCATTCAATTGCCA |
| ISG15 | | GTGGACAAATGCGACGAACC | ATTTCCGGCCCTTGATCCTG |
| OAS1 | | GAGCTCCTGACGGTCTATGC | TCATCGTCTGCACTGTTGCT |
| Mx1 | | TTTCAAGAAGGAGGCCAGCAA | TCATGTGCATCTGAGGGTGG |
| TRIM54 | | GGCCTCATCCGTCAGTATGG | CCTTGGCCTGCTGGAGATAC |
| SeV HN | | GCTTACGGGACAGATGAGAT | ATTGTTATGAACCGACTTGC |
| DENV E | CATTCCAAGTGAGAATCTCTTTGTCA | | CAGATCTCTGATGAATAACCAACG |
| HPRT | | AGGACTGAACGTCTTGCTCG | ATCCAACACTTCGTGGGGTC |
